# Modeling of LCF Behaviour on AISI316L Steel Applying the Armstrong–Frederick Kinematic Hardening Model

**DOI:** 10.3390/ma17143395

**Published:** 2024-07-09

**Authors:** Sushant Bhalchandra Pate, Gintautas Dundulis, Paulius Griskevicius

**Affiliations:** Faculty of Mechanical Engineering and Design, Kaunas University of Technology, K. Donelaičio g. 73, 44249 Kaunas, Lithuania; gintautas.dundulis@ktu.lt (G.D.); paulius.griskevicius@ktu.lt (P.G.)

**Keywords:** low-cycle fatigue, AISI316L steel, FE numerical investigation

## Abstract

The combination of kinematic and isotropic hardening models makes it possible to model the behaviour of cyclic elastic-plastic steel material, though the estimation of the hardening parameters and catching the influence of those parameters on the material response is a challenging task. In the current work, an approach for the numerical simulation of the low-cycle fatigue of AISI316L steel is presented using a finite element method to study the fatigue behaviour of the steel at different strain amplitudes and operating temperatures. Fully reversed uniaxial LCF tests are performed at different strain amplitudes and operating temperatures. Based on the LCF test experimental results, the non-linear isotropic and kinematic hardening parameters are estimated for numerical simulation. On comparing, the numerical simulation results were in very good agreement with those of the experimental ones. This presented method for the numerical simulation of the low-cycle fatigue on AISI316 stainless steel can be used for the approximate prediction of the fatigue life of the components under different cyclic loading amplitudes.

## 1. Introduction

Environmentally assisted fatigue (EAF) significantly affects the fatigue life (N_f_) of components operating in pressurized water reactor (PWR) environments. The components operating in such environments are manufactured using austenitic stainless steel.

The importance of this degradation mechanism is shown in the European Commission’s initiation of the H2020 projects for the research of EAF. In 2015–2020, the European Commission initiated a H2020 project entitled Increasing Safety in Nuclear Power Plants by Covering Gaps in Environmental Fatigue Assessment (INCEFA-PLUS). In this project, experimental LCF tests of 304 steels were performed [1], applying test conditions that mimicked those of the NPP while maintaining realistic test durations. In 2020, another project entitled ‘Increasing safety in NPPs by covering gaps in Environmental Fatigue Assessment’, focussing on gaps between laboratory data and components under SCALE (INCEFA-Scale), emerged as a successor of the INCEFA-Plus project. The main goal of this project is to continue work, advancing the ability to predict the lifetimes of nuclear plant components when subjected to Environmental Assisted Fatigue (EAF). The main issue addressed by INCEFA-SCALE is the transferability of laboratory-scale tests to real nuclear components [2,3,4]. In this project, 316L steel was selected as a common material for the experimental LCF tests.

The AISI316L steel is widely used for the design of the components that operate under extreme working conditions, such as high temperatures, temperature gradients and alternative loadings. These components are exposed to mechanical and/or thermal cyclic strain and stresses, due to which thermo-mechanical fatigue can be observed. This leads to crack initiation followed by crack growth, which generally occurs in the fields of power, energy industries such as in the LMFBR (liquid metal-cooled fast breeder reactors), nuclear reactors, pressure vessels, and the aerospace industry. Low-cycle fatigue damage can be observed as a dominant failure mode which occurs during the start and shut down cycles of the nuclear reactors and requires the attention during the design and life analysis of the components [5,6]. Low-cycle fatigue loads are the significant factors leading to the failure of critical components of aeronautics and power plant structures [7]. Accurate estimation of the operational life of the components is a very important condition for the design and maintenance of these components to reduce the risk of sudden breakdown of the plant [7].

Hongyan Duan et al. [8,9] applied different machine learning techniques to predict low-cycle fatigue life of 316 austenitic stainless steel. The results show that the prediction accuracy is sufficient for engineering applications, where some of the models had prediction accuracy R2 up to 93%. The studies shows that appropriately created deep learning models allow with appropriate accuracy to evaluate the effect of strain amplitude, temperature and heat treatment holding time on the low-cycle fatigue life. Machine learning-based damage models proposed by Michal Bartosak [10] showed good agreement between experimental results and predictions for low-cycle fatigue lifetime under complex thermo-mechanical loading.

There has been extensive research in the field of experimental and/or numerical investigation of low-cycle fatigue of the steel components operating under extreme conditions. Yinfeng C et al. [5], investigated the low-cycle fatigue properties of additively manufactured 316 stainless steels by comparing the results with the fatigue properties of rolled 316 SS parts. R. Hormozi et al. [6], performed an experimental investigation of a fully reversed isothermal fatigue test for several strain ranges at 650 °C on 316 steels along with a numerical investigation using the isotropic and non-linear kinematic hardening rule to duplicate the behaviour of the experimental material until the stabilisation of the component. Ondrej S et al. [7], investigated the low-cycle fatigue behaviour of the AISI316L austenitic steel using experimental and numerical investigation methodology, exposed to cyclic loading in the hollow cylindrical specimen with pre-existing crack on the specimen. Roy S et al. [11], predicted the low-cycle fatigue life of AISI316L stainless steel at room temperature. They performed several strain-controlled low-cycle fatigue experimental tests for strain ranging from 0.3% to 1% strain with a strain rate of 0.3%/s and performed numerical investigation using finite element analysis employing Choboche kinematic hardening model with the isotropic hardening to predict the fatigue life of the specimen. Moeini G et al. [12], investigated experimentally and numerically the low-cycle fatigue behaviour of dual-phase steel. They simulated the stabilised hysteresis loop employing micro-mechanical modelling. Branco R et al. [13], performed fully reversed strain-controlled low-cycle fatigue experiments for the strain amplitude between 0.3 and 0.1% strain on AISI 18Ni300 steel at room temperature. Wang X et al. [14], investigated the effects of hold time, strain amplitude, and temperature on the fatigue life of P92 steel by performing several low-cycle fatigue experiments on it and predicted the fatigue life of several experiments performed. Vaitkunas T et al. [15], performed low-cycle fatigue test on the 316L stainless steel specimen which was followed by KTF-PD (kinetic theory of fracture-peridynamics) simulation and proposed novel KTF equation for predicting fatigue life. Zhu P et al. [16], has proposed the hardening function and investigated the factors affecting the hardening. H. Mahbadi and M.R. Eslami [17] employed the Prager and Armstron-Frederick kinematic hardening models for the investigation of cyclic loading.

Despite extensive research carried out on the kinematic hardening models; it remains a complex process of estimating pairs of kinematic hardening parameters that can capture proper elastoplastic behaviour of the steel. The aim of the presented research is to continue an approach to estimate kinematic hardening parameters for numerical investigation of the low-cycle fatigue behaviour of AISI 316L stainless steel.

The main task of this manuscript is the application of the finite element method for numerical simulation to investigate the low-cycle fatigue. In this research, to investigate the low-cycle fatigue behaviour of AISI 316L stainless steel, strain-controlled low-cycle fatigue experiments and numerical simulations were performed for 0.18% and 0.6% strain amplitudes at room temperature and an elevated temperature of 300 °C, respectively. To model the material behaviour of the steel for numerical simulation, a combination of isotropic and kinematic hardening was employed. The required material parameters were estimated using the recorded experimental data; this is explained in the following sections. The efficiency of the estimated parameters is tested by performing a numerical simulation using finite element software, LS-Dyna, and comparing the simulation results with the experimental ones. The Armstrong–Frederick kinematic hardening model was employed to define the kinematic hardening behaviour of the material. The Armstrong and Frederick kinematic hardening model suits the scheme of the presented research as this model is able to predict the plasticity for strain-controlled cyclic loading with zero mean stress. The simulation results were validated by comparing them with the results of the experimental test. 

## 2. Materials and Methodology

### 2.1. Experimental Low-Cycle Fatigue Test

Strain-controlled LCF tests on AISI316L stainless steel were performed under the Horizon 2020 INCEFA-Scale project. Fatigue tests were used to study the effects of complex loading (variable amplitudes), the strain range, the environment, and temperature on the fatigue life of 316L stainless steel subjected to an applied strain range (or occasional stress range) for a relatively low number of cycles (low-cycle fatigue) [2,3]. INCEFA-SCALE modelling plans and some illustrations on the five main topics that have been identified: (1) numerical analyses to support test design and interpretation, (2) data mining, (3) a review of codified methods, (4) fatigue damage modelling and non-codified approaches to better address fatigue damage mechanisms, and (5) industrial applications [4].

#### 2.1.1. Specimens and Materials

**Figure 1:**
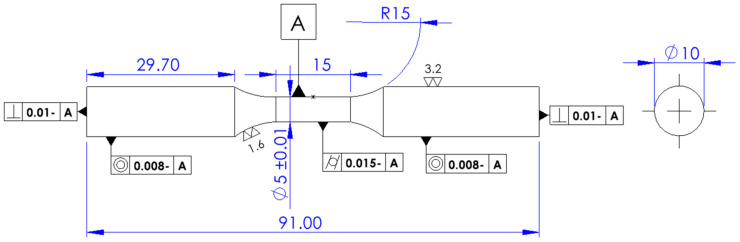
A drawing of the LCF experimental test specimen.

The specimen was prepared according to the drawing presented in Figure 1. The dimensions of the test specimens were decided according to ASTM E606/E606M [18]. The nominal specimen gauge length and the extensometer gauge length were 15 mm and 12.5 mm, respectively. The diameter of the specimen was 5 mm. The surface of the specimen was polished for an Ra of 1.6.

Table 1 represents the material properties of AISI316L stainless steel.

#### 2.1.2. LCF Experimental Test

Cyclic tests were carried out on the universal testing machine (ElectroPuls E10000, Instron, Norwood, MA, USA). The experiment was carried out at room temperature at 20 °C (293 K). Strain-controlled fatigue tests were run in symmetric triangle waveform constant-amplitude cycles with ε_max_ = 0.18% and ε_max_ = 0.6% strain amplitudes at a frequency of 0.56 Hz. The measured fatigue life was 342,870 cycles and 1183 cycles, respectively. The failure was defined at a point when the maximum stress decreased by 25%.

### 2.2. Numerical Simulation of LCF

To study the fatigue behaviour of AISI316L steel through numerical simulation, the finite element method was employed. The simulations were performed using FE software LS-Dyna R13.1.0. LS-Dyna employs an Armstrong–Frederick kinematic hardening model to define the kinematic hardening behaviour of the material.

#### 2.2.1. Finite Element Modelling

The finite element model of the specimen was prepared referring to the drawing of the experimental test specimen (Figure 1). Only a quarter portion of the gauge length of the experimental specimen was considered for the numerical simulation due to the axisymmetry of the specimen.

**Figure 2:**
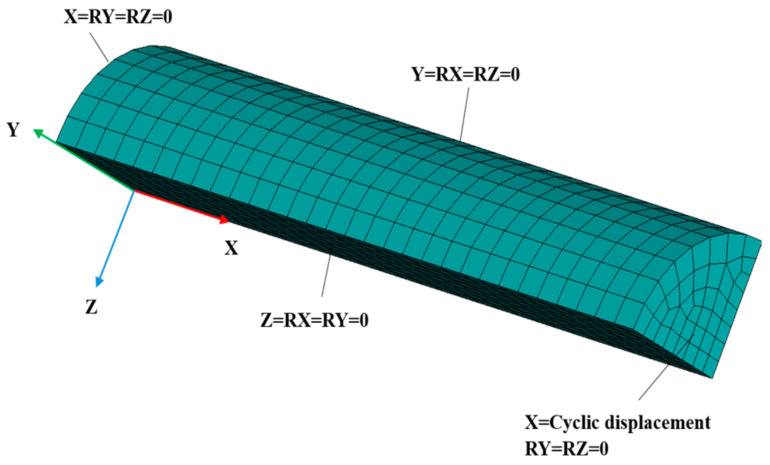
The meshed finite element model with the applied boundary conditions.

Figure 2 represents the finite element model and the boundary conditions of the finite element model for the LCF simulation. The FE model was prepared employing eight-node hexahedron constant stress solid elements. The model consisted of 1200 elements with a size of 0.5 mm. Each node of these elements had a three-dimensional degree of freedom.

**Figure 3:**
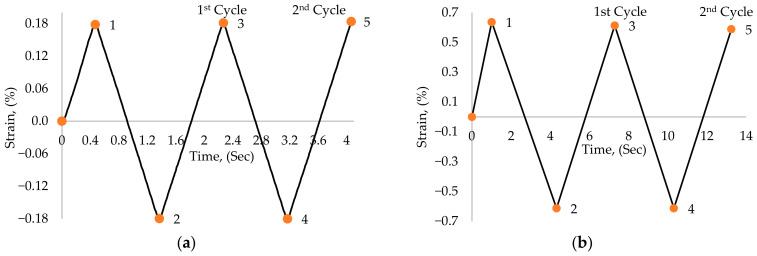
The relative displacement for specimen loading (**a**) for 0.18% strain at RT, and (**b**) for 0.6% strain at 300 °C.

For the modelling of the expected displacement of the model, required boundary conditions were applied to the specimen model (Figure 2). The flat surface of the specimen was exposed to cyclic displacement and the motion of the other flat faces was constrained as per Figure 3.

#### 2.2.2. Specimen Loading

Constant-amplitude fully reversed cyclic displacement in a triangular waveform, as per Figure 3, was applied to the model along the X-axial direction to mimic the strain-controlled cyclic motion, which can be observed in Figure 2.

Figure 3 represents the applied strain (%) versus the time (Sec) used to apply it. Figure 3a represents the applied strain of the 0.18% strain amplitude model and Figure 3b represents the applied strain for the 0.6% strain amplitude. For both specimens, the first cycle initiates from the rest position of the specimen (i.e., 0% strain) and proceeds until 1, which is a quarter of one full cycle, followed by the path (1–2) compression part of the cycle and (2–3) tension part of the cycle to complete the first cycle. The second cycle follows the path (3–4–5), and so on.

#### 2.2.3. Material Modelling

An important consideration that should be taken into account during the finite element modelling of components under LCF is the selection of the correct elastoplastic material model [11]. The selected material model should be able to capture the behaviour of the material as closely as possible to that of the experimental results [11]. To model the material for numerical simulation, isotropic hardening along with kinematic hardening was employed. Ls-Dyna has a predefined material model titled MAT_DAMAGE_3. This material model consists of kinematic hardening combined with the isotropic hardening for modelling low-cycle fatigue.

The isotropic hardening applied to the numerical model was calculated on the experimental results recorded from the low-cycle fatigue test.

**Figure 4:**
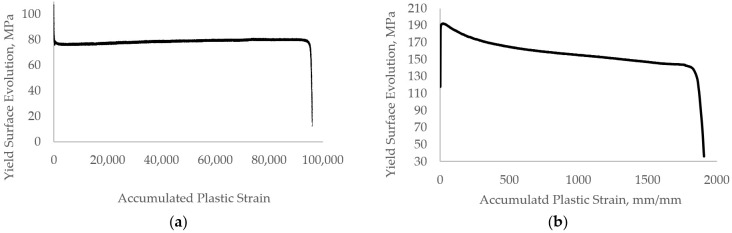
The isotropic hardening curve used in LS-Dyna for the (**a**) 0.18% strain model, and the (**b**) 0.6% strain model.

Figure 4 represents the isotropic hardening curve applied to the numerical simulation. The yield surface evolution (σ) versus accumulated plastic strain $\left({\bar{\varepsilon }}_{p}\right)$ curve is plotted for the isotropic hardening curve. The curve presented in Figure 4a,b is used to describe the material models of 0.18% strain and 0.6% strain, respectively.

The evolution of the yield stress was calculated according to Equation (1).

(1) $$\sigma =\sigma_{max,i}-\sigma_{y0}\;$$

$\sigma_{max,i}$: Maximum stress for the current cycle.
$\sigma_{y0}$: Initial Yield Stress

Equation (2) represents the accumulated plastic strain.

(2) $${\dot{\bar{\varepsilon }}}^{pl}={2N∆\varepsilon }_{pl}$$

*N*: number of cycles.
${∆\varepsilon }_{pl}$: Plastic strain range (%)

Kinematic hardening material models translate the loading surface as a rigid body in the stress space while keeping the initial yield surface size, shape, and orientation. There have been several kinematic hardening models proposed in the past. Prager proposed the linear kinematic hardening model, in which he assumed a collinear relationship between the plastic strain increment and the kinematic variable increment [17]. The Prager model gives an identical strain hardening curve in the tension and compression part of the cyclic loading, but it may not be the same for the experimental data [17]. Armstrong and Frederick proposed a non-linear kinematic hardening model in which they eliminated the proportionality between the plastic strain increment and the kinematic variable increment from the Prager model to obtain a smooth transition between the elastic and plastic region [17]. Chaboche superimposed several Armstrong and Frederick nonlinear kinematic hardening models and proposed a new nonlinear kinematic hardening model to describe a stable hysteresis curve more accurately.

In the presented research, the Armstrong and Frederick nonlinear kinematic hardening model was employed. The Armstrong–Frederick kinematic hardening model is broadly employed for the simulation of the hardening behaviour of the steel. The advantage of this model is that the total backstress of the stress–strain curve is divided into fewer parts and each part has a limited surface. This limiting surface concept provides a convenient framework to construct refined hardening algorithms [19].

The MAT_DAMAGE_3 material model in LS-Dyna uses the Armstrong–Frederick kinematic hardening model.

The Armstrong–Frederick kinematic hardening model is defined in LS-Dyna as Equation (3).

(3) $${\dot{\alpha }}_{j}=\frac{2}{3}C_{j}{\dot{\varepsilon }}^{pl}-\gamma_{j}\alpha_{j}{\dot{\bar{\varepsilon }}}^{pl}$$

*C*: Coefficient of Kinematic Hardening (MPa)
γ: Exponent for Kinematic Hardening
${\dot{\varepsilon }}^{pl}$: Plastic strain
${\dot{\bar{\varepsilon }}}^{pl}$: Accumulated Plastic Deformation
$\alpha_{j}$: Backstress

The kinematic hardening parameters were estimated according to Equation (4). Three significant points on the tension-loading side of the stress–strain hysteresis loop of the stabilised cycle 171,000 and cycle 595 were selected for the 0.18% and 0.6% strain models, respectively.

(4) $$\alpha_{max}=\sum_{j=1}^{3}\left[\frac{2}{3}C_{j}{\dot{\varepsilon }}^{pl}-\gamma_{j}\alpha_{j}{\dot{\bar{\varepsilon }}}^{pl}\right]$$

$\alpha_{max}$: Maximum cycle stress (MPa)

The estimated KH parameters are presented in Table 2; these parameters were employed for the low-cycle fatigue simulation in LS-Dyna.

## 3. Results

### 3.1. LCF Experiment

**Figure 5:**
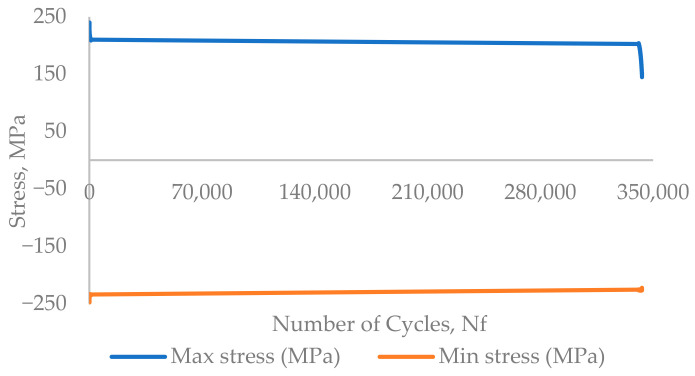
The experimental results of the 0.18% strain amplitude, T = 20 °C, stress versus number of cycles curve.

**Figure 6:**
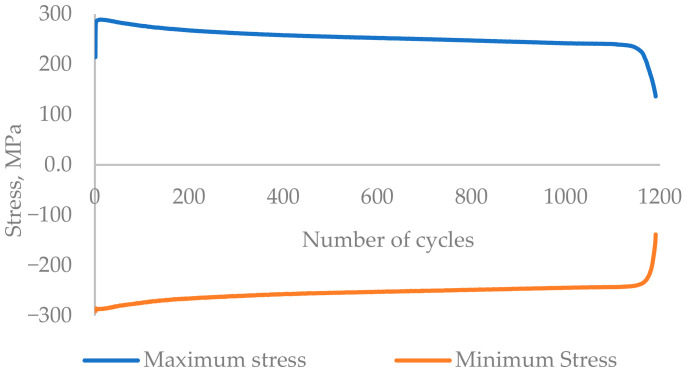
The experimental results of the 0.6% strain amplitude, T = 300 °C, stress versus number of cycles curve.

**Figure 7:**
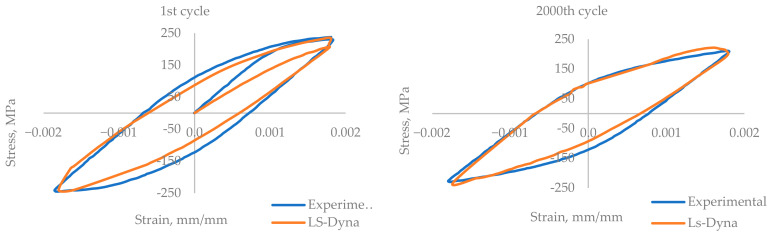
A comparison of the stress-versus-strain hysteresis for the 1st and 2000th cycles of the 0.18% strain model.

**Figure 8:**
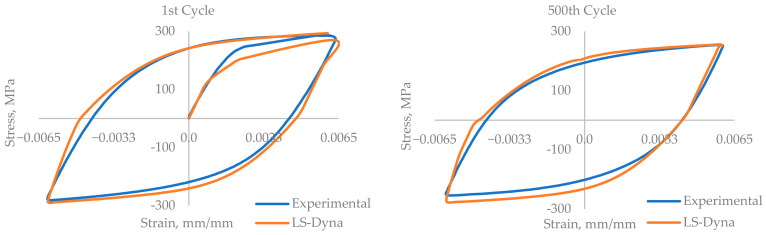
A comparison of the stress-versus-strain hysteresis for the 1st and 500th cycles of the 0.6% strain model.

The experimental results are presented in the form of a curve of plotted stress versus the number of cycles for the whole experiment, Figure 5 and Figure 6. The experiments were performed until the failure of the component. Figure 7 and Figure 8 show the cyclic stress versus strain curves for the 1st and 2000th cycles for the 0.18% strain amplitude experiment and for the 1st and 500th cycles for the 0.6% strain amplitude experiment.

The failure of the specimen was considered in loading cycle 343,106 for 0.18% strain and loading cycle 1192 for 0.6% strain, where the stress amplitude drops by 25% with respect to the maximum stress level. 

### 3.2. LCF Simulation

For the numerical simulation of the low-cycle fatigue behaviour of the component subjected to cyclic loading, the cyclic plasticity of the material should be modelled until the results reach stabilisation [20]. The finite element method is a convenient tool for predicting the stress–strain history to estimate the fatigue life of the component as closely as possible to the actual fatigue life of the component [11]. The durability of components that undergo cyclic loading can be estimated if a numerical simulation is performed until it reaches the stabilisation state, with the stabilisation of the material generally being observed at the halfway point of the total number of loading cycles until the failure of the part [11].

The simulation was performed by employing the explicit solver (default) in Ls-Dyna R13.1.0 software. The results of the numerical simulation were plotted as the stress-versus-strain hysteresis loop in Figure 7, and the maximum stress for the cycle versus the number of cycles in Figure 8, along with the experimental results.

For the comparison of the numerical simulation results and the experimental results, the hysteresis loop was plotted for stress versus strain. The hysteresis loop was plotted for the 1st and 2000th cycles, presented in Figure 7. Upon comparing the hysteresis loops, they showed very close agreement. Slight deviation in the elastic and plastic stress–strain intersection point is observed between the experimental and simulation results. The maximum stress obtained from the model is higher than the experimental value.

**Figure 9:**
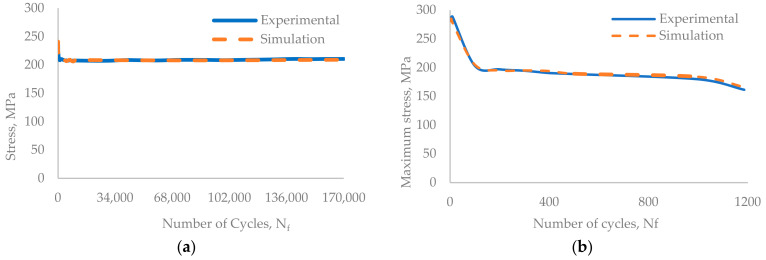
A comparison between the experimental and simulation stress-versus-number-of-cycle results of (**a**) 0.18% strain and (**b**) 0.6% strain.

To check the validity of the numerical model prepared by applying isotropic hardening along with kinematic hardening to model the material for the total number of loading cycles up to the failure, the results obtained from the numerical simulation were compared with the experimental results. For the same purpose, a curve was plotted for the maximum stress versus the corresponding number of cycles, which is presented in Figure 9.

**Figure 10:**
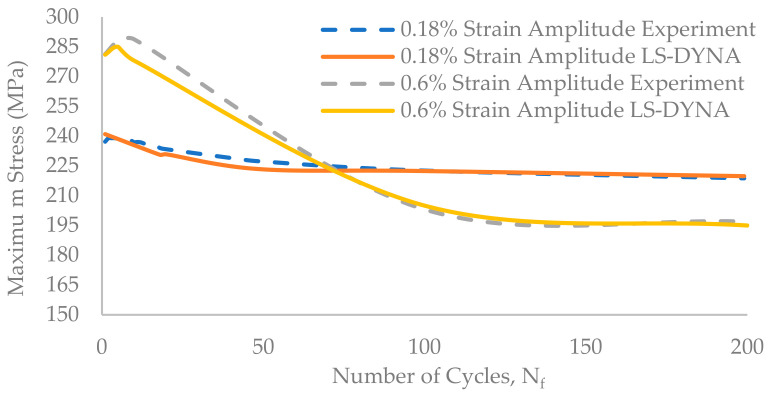
A comparison between experimental and simulation stress-versus-number-of-cycle results for the initial 200 cycles.

The simulation results agreed well with the experimental results. For the initial few cycles, the maximum stress value deviates from the experimental results. For the 0.18% strain amplitude model, the maximum stress deviates by 1.58% between the 1st and the 100th cycle and by 0.5% between the 101st and the 170,000th cycle. In the case of the 0.6% strain amplitude model, the deviation was 3.7% for cycles between the 1st and the 60^th^ and 1% from the 61st to the 1192nd cycles. The proposed numerical simulation model captures the cyclic hardening for initial cycles followed by the cyclic softening behaviour that is similar to the experimental results (Figure 10). The proposed model can be used for the approximate prediction of the fatigue life of the component exposed to similar loading conditions.

## 4. Discussion

The low-cycle fatigue experimental results recorded for the 0.18% strain amplitude at room temperature showed an increase in the maximum stress of each cycle until the 6th cycle. From the 7th cycle, the maximum stress per cycle decreases until the 1235th cycle, followed by a kind of stabilised region with the range of stress between 210 MPa and 214 MPa until the 326,557th cycle. After the 326,557th cycle, the maximum stress decreases continuously for consecutive cycles until the failure of the specimen, i.e., the fracture which occurred in the 342,870 cycle. In the results of another experiment performed on an AISI316L stainless steel component at 300 °C with a 0.6% strain amplitude show a similar behaviour: the maximum stress for the initial cycles increases until the 10th cycle, and then the maximum stress continuously decreases until the failure of the component, which occurs in the 1183rd cycle. No stabilised region was observed in the 0.6% stain amplitude experiment, and neither was one observed in the results of the 0.18% strain amplitude experiment. A common behaviour of a reduction in the maximum stress after an increase in stress for the initial few cycles is observed in both experiments’ results. This shows that AISI316L stainless steel exhibits a softening-of-the-material behaviour when exposed to cyclic loading.

The numerical simulation of the low-cycle fatigue test was performed for the 0.18% strain amplitude and the 0.6% strain amplitude, and the results are plotted and compared with the experimental results. For the experiments and simulations, the results are plotted for the maximum stress of the cycles versus the number of cycles and the cyclic stress–strain curve. For the stress–strain hysteresis curve of the 1st cycle, there is deflection in the shape of the curve, but the shape gets better as the simulation progresses, and for the 2000th cycle of 0.18% strain and the 500th cycle of 0.6% strain, the hysteresis loop gets better and close to the experimental results. The results of the numerical simulation are very close to those of the experiments, with a maximum deflection of 1.58% and 3.7% in the maximum stress level for the 0.18% strain amplitude experiment and the 0.6% strain amplitude experiment, respectively.

## 5. Conclusions

In the presented research, strain-controlled low-cycle fatigue tests for strain amplitudes of 0.18% and 0.6% at room temperature and 300 °C, respectively, are performed on AISI 316L stainless steel. The experimental results are employed to estimate the material properties and parameters required for the numerical modelling of the material for the low-cycle fatigue simulation. The combination of isotropic hardening and kinematic hardening is used for the modelling of the material to capture the expected behaviour of the material during the numerical simulation.

The results are plotted in the form of curves of the stress versus strain and the maximum stress versus the corresponding number of cycles. The simulation results are validated by comparing them with the experimental results.

By comparing the results, it is observed that the simulation results curve matches up well with the experimental results curve. For the initial cycles, there was deviation between the experimental and simulation results, but as the loading cycles proceeded, the simulation results got closer to the experimental values.

The finite element methodology can be employed for the numerical simulation of AISI316L stainless steel under constant amplitude, repetitive, strain-controlled low-cycle fatigue behaviour. The approximate fatigue life of stainless steel components operating under similar conditions can be estimated using this methodology.

## Figures and Tables

**Table 1:** The material properties AISI316L steel.

Quantity	Temperature	Young’s Modulus	Poisson’s Ratio	Yield Stress	Ultimate Tensile Strength
Symbol	T	E	υ	$\sigma_{y}$	$\sigma_{uts}$
Units	°C	GPa	-	MPa	MPa
Values	20	201	0.3	133	554
	300	160	0.27	68	439

**Table 2:** The kinematic hardening parameters used in the Ls-Dyna.

**Strain Amplitude**	**Temperature, °C**	**C1, MPa**	**ϒ** **1**	**C2, MPa**	**ϒ** **2**	**C3, MPa**	**ϒ** **3**
0.18%	20	150,500	1680	450,650	2530	10,560	1360
0.6%	300	5000	83.3	100,000	825	1000	1530

## Data Availability

Requests to access the data presented in this study can be submitted to the data owner(s). The data are stored in a database and traceable through DOIs (MatDB(JRC)) but are not publicly available due to the data policy of the project.
